# Selecting Microbial Strains from Pine Tree Resin: Biotechnological Applications from a Terpene World

**DOI:** 10.1371/journal.pone.0100740

**Published:** 2014-06-27

**Authors:** Cristina Vilanova, Maria Marín, Joaquín Baixeras, Amparo Latorre, Manuel Porcar

**Affiliations:** 1 Universitat de València (Cavanilles Institute of Biodiversity and Evolutive Biology), Valencia, Spain; 2 Unidad Mixta de Investigación en Genómica y Salud, Centro Superior de Investigación en Salud Pública (CSISP), València, Spain; 3 Fundació General de la Universitat de València, València, Spain; International Atomic Energy Agency, Austria

## Abstract

Resin is a chemical and physical defensive barrier secreted by many plants, especially coniferous trees, with insecticidal and antimicrobial properties. The degradation of terpenes, the main components accounting for the toxicity of resin, is highly relevant for a vast range of biotechnological processes, including bioremediation. In the present work, we used a resin-based selective medium in order to study the resin-tolerant microbial communities associated with the galls formed by the moth *Retinia resinella*; as well as resin from *Pinus sylvestris* forests, one of the largest ecosystems on Earth and a yet-unexplored source of terpene-degrading microorganisms. The taxonomic and functional diversity of the cultivated, resin-tolerant fraction of the whole microbiota were unveiled by high-throughput sequencing, which resulted in the detection of more than 40 bacterial genera among the terpene-degrading microorganisms, and a range of genes involved in the degradation of different terpene families. We further characterized through culture-based approaches and transcriptome sequencing selected microbial strains, including *Pseudomonas* sp., the most abundant species in both environmental resin and *R. resinella* resin-rich galls, and three fungal species, and experimentally confirmed their ability to degrade resin and also other terpene-based compounds and, thus, their potential use in biotechnological applications involving terpene catabolism.

## Introduction

Coniferous resin is a complex mixture of secondary metabolites. Resin protects injured tree tissues from phytophagous insects [1] and plant pathogens [2], [3], [4], [5]. Terpenoids, flavonoids, and fatty acids are the main components of resin [6], [7]. Among these, terpenes (terpenoids containing a variable number of complete repetitions of isoprene) are the best characterized metabolites because they can be easily identified with techniques such as gas chromatography. Monoterpenes, sesquiterpenes, and diterpenes (containing two, three, and four repetitions of isoprene, respectively) are the most abundant families of terpenes in pine tree resin [6]. Since most of these *de novo* synthesized compounds display antibacterial and antifungal properties, they are considered to be phytoalexins accounting for the toxicity of resin [8], [9], [10], [11].

Many terpenes display interesting features for the chemical industry, specifically in the production of fragrances, essential oils, and food additives [12]. They are also valuable molecules in medicine because of their cytotoxic, cardiotonic, and anti- inflammatory properties [13], [14]. However, terpenes are one of the main pollutants in effluents of pulp mill industries [15], [16], and terpene-based materials such as tire rubber or latex account for tons of solid waste per year. Thus, these molecules are among the main chemical targets for bioremediation [17]. Given all their applications in biotechnology, bioprospection aiming to identify single genes, gene networks, and microorganisms able to transform or catabolize these molecules is a key starting point in the development of a range of biotechnological applications. The toxicity of terpenes and complexity of their chemical structure hinder their degradation by microorganisms, and few studies describe the ability of microorganisms to use individual components of resin as a sole carbon source [18] or to biotransform particular terpene molecules [19]. The ability of some insects to overcome tree's defensive compounds [20] and terpenes in particular [21] has been previously attributed to their association with microorganisms.

A yet unexplored source of potential terpene-degrading microorganisms is related to the insect *Retinia resinella* Linnaeus. The larvae of *R. resinella* feed on young twigs of the Scotch pine *Pinus sylvestris* [22], [23]. They cause small wounds that induce the secretion of resin, which is manipulated by the larva to construct a nodule-like resin capsule –commonly known as “resin gall”- as a hard protective cocoon. A single larva develops inside this resin blister for nearly two years, completely isolated from the external environment by its terpene-rich shelter [24]. *R. resinella*'s gut microbiota has not been studied to date, and represents a potential reservoir of resin-tolerant microorganisms. This work, however, aimed at selecting and identifying the cultivable microbial communities associated to *P. sylvestris* resin and *R. resinella* resin-rich galls with a potential ability to degrade terpenes. In order to do so, we used a holistic approach starting from strain selection on a resin-containing medium; genomic and transcriptomic analyses and microbial confrontation assays. The combination of high-throughput sequencing, with the selection of bacterial and fungal strains with outstanding terpene degradation ability, enabled us to characterize natural isolates with promising biotechnological applications.

## Materials and Methods

### Sample collection

Samples of Scot pine (*Pinus sylvestris*)-associated environmental resin -taken from lopped trees-, and *R. resinella* galls were collected from different trees covering an area of about 1 km^2^ along the forest trail “Fuente del Tajo” (N 40° 17′, W 0° 36′), Mora de Rubielos, Teruel, Spain) from September 2011 to March 2012. Oficial permissions for collecting were provided by INAGA (Instituto Aragonés de Gestión Ambiental). Larvae were removed from the galls, and both galls and resin coming from different trees were pooled and separately ground with a porcelain pestle to obtain small particles of 4–5 mm. The associated microbiota was harvested by washing 10 g of these particles in sterile PBS buffer (NaCl 8 g/L, KCl 0.2 g/L, Na_2_HPO_4_ 1.44 g/L, KH_2_PO_4_ 0.24 g/L, pH adjusted to 7.4), obtaining a suspension that was subsequently cultured in selective media.

### Culture media and growth conditions

A selective minimal medium containing pine resin was employed. As the selection factor, a 10% (w/v) stock solution of resin was obtained by dissolving resin samples in absolute ethanol. Plant debris and other insoluble particles in suspension were eliminated by centrifugation (2,000xg, 5 min) and the supernatant was filter-sterilized through a 0.2 µm pore diameter filter (Corning Inc., NY, USA). A minimal medium was prepared (2 g/L NaNO_3_, 1 g/L K_2_HPO_4_, 0.5 g/L MgSO_4_, 0.5 g/L KCl, 0.2 g/L bacteriological peptone; and 15 g/L agar for solid medium). Once sterilized by autoclave, this minimal medium was kept at 80°C and mixed with the resin:ethanol stock solution (ethanol evaporated under these conditions), yielding a selective medium, hereafter called RM (Resin-based Medium), with increasing resin concentrations (0.05%, 0.1%, 0.2%, 0.4%, and 0.8% w/v) as the main carbon source.

Pine tree resin (from healthy pine wounds) and galls suspensions, including small environmental resin and galls resin particles, were spread on the selective medium plates (in 5 replica for each resin concentration) and incubated at 30°C for 14 days.

### DNA extraction

Microbial colonies observed on the plates of increasing resin concentrations were harvested by washing the plates with sterile PBS. Each plate was washed using the same volume of PBS, and the resulting suspensions were pooled in two independent tubes in order to isolate the total DNA of the communities cultivated from galls and environmental resin, respectively. The Power Soil DNA Isolation kit (MO BIO Laboratories) was used following the manufacturer's instructions with an additional pretreatment with DNA-free lysozyme at 37°C for 10 min. The quantity and quality of the DNA was determined on a 1.5% agarose gel and with a Nanodrop-1000 Spectophotometer (Thermo Scientific, Wilmington, DE).

### DNA sequencing, assembly and ORF prediction

Two shotgun libraries were created from 1 µg of the total DNA of the communities cultivated from galls and environmental resin, respectively, according to manufacturer instructions (Roche, Rapid Library Preparation Method Manual GS FLX+ Series XL+, May 2011). Average insert size was 1,800 bp. Each library was sequenced at the CSISP (Centro Superior de Investigación en Salud Pública, Valencia, Spain) using a half pyrosequencing plate in a Roche 454 FLS GS Titanium sequencer. The sequences obtained were assembled using the NEWBLER software (454 LifeSciences Roche) with the default parameters, and the resulting assembly was manually revised and curated with the software Gap4 of the Staden Package [25]. Finally, a prediction of Open Reading Frames (ORFs) was carried out on the assemblies using the MetaGeneAnnotator program [26], based on Hidden Markov Models (HMMs). Predicted ORFs smaller than 100 bp were not considered for further analysis.

All sequences were deposited and made publicly available in the MG-RAST server with the following accession numbers: 4455198.3 and 4454707.3.

### Sequence analysis

Taxonomic assignations were performed by combining different methods based on sequence similarity. In a first approach, sequences from the 16 S and 18 S ribosomal RNA genes were used as phylogenetic markers, but only a few sequences of this type were found among both the unassembled and the assembled reads due to the high number of sequences corresponding to *Pseudomonas* sp., the majority species in both metagenomes. In order to improve the detection of other taxa, an alternative protein-based taxonomic binning was performed. First, unassembled reads from each cultivated community were analyzed with the MG-RAST server, based on the SEED framework [27], and taxonomic assignations were obtained based on sequence similarity searches. Only assignments with an e-value less than or equal to 10^−5^ and a similarity percentage greater than or equal to 80% were accepted. Second, an *ad hoc* sequence analysis pipeline was used. The analysis consisted of BLASTX searches against the non-redundant protein division of GenBank with the ORFs predicted from the assemblies. Again, an e-value of 10^−5^ was used as threshold. BLASTX results were processed with the MEGAN software [28], assuming a direct correlation between the number of reads corresponding to a particular taxon and the number of ORFs found for it. The more abundant a taxon, the more reads it generates, resulting in longer contigs (Figure S1) and a higher number of ORFs identified. Finally, the putative taxonomic assignments obtained for the most abundant species according to the above-described methods were revised and confirmed by analyzing the coding sequences of housekeeping genes extracted from the assemblies. To do this, *16 S*, *rpoD* and *gyrB* sequences of the NCBI nucleotide database belonging to different species of the most abundant genera were retrieved and aligned using software MEGA. Then, a phylogenetic reconstruction of the sequences was made, and taxonomic assignations were performed accordingly.

In order to obtain functional information of the predicted ORFs from each sample, BLASTP searches were performed against the COG (Cluster of Orthologous Groups of proteins) database [29]. Metabolic reconstructions based on gene content were obtained with the program KAAS through BLASTP searches against the KEGG Genes database. In all cases, only hits with an associated e-value less than or equal to 10^−5^ were kept. In addition, the functional assignments provided by the MG-RAST server, based on searches against the non-redundant protein subdivision of GenBank, were also considered for the functional annotation of the ORFs.

### Isolation of microbial strains in pure cultures

*Pseudomonas* strain PS was isolated from a Petri plate of selective medium (containing a concentration 0.1% w/v of resin) where a galls suspension had been spread and that had been incubated for 14 days at 30°C. Several fungal strains were isolated from other plates (containing different amounts of resin −0.05%, 0.1%, and 0.2% w/v-) where environmental resin samples had been cultured under the same conditions. In all cases, individual colonies were picked and two consecutive re-isolations were performed in resin selective medium (0.1% w/v). Then, pure cultures were set up in resin selective liquid medium. For the cryopreservation of bacteria, a stock solution of glycerol 50% was prepared by mixing equal volumes of glycerol (Panreac Química S.L.U., Barcelona, Spain) and sterile water. Aliquots from each liquid culture were stored in 500 mL of this solution at −20°C until required.

### Fungal inhibition assays

The antifungal properties of the *Pseudomonas* sp. isolate were tested with a confrontation assay following the procedure previously described [30]. As a control, all the isolates were grown alone, under the same conditions but without *Pseudomonas* confrontation. All confrontation assays were performed in both RM (0.1% resin w/v) and LB solid media. The fungal growth inhibition was assessed by comparing the diameter of the confronted *vs* the isolated fungal colonies at different times and in three independent replicas.

### Total RNA isolation, mRNA amplification and cDNA synthesis

To perform the transcriptomic analysis, confronted and isolated fungal colonies from three independent replica of the confrontation assays were separately pooled, and total RNA was extracted with TRI Reagent solution (Ambion). The quantity and the integrity of the total RNA was determined on a 0.8% agarose gel and with a Nanodrop-1000 Spectophotometer (Thermo Scientific, Wilmington, DE).

Then, mRNAs were amplified with MessageAmp II aRNA Amplification Kit (Ambion). The resulting RNA was converted to double-stranded cDNA using random hexamers. Again, the quantity and quality of the cDNA was assessed on a 0.8% agarose gel and with a standard PicoGreen Assay for dsDNA (Thermo Scientific, Wilmington, DE).

### cDNA sequencing and assembly

The cDNA of both confronted and isolated fungal samples was sequenced in a Roche 454 FLS GS Titanium sequencer, using 1/8 of a pyrosequencing plate for each sample. The resulting reads were trimmed and assembled with the NEWBLER software (454 LifeSciences Roche), using the default parameters for cDNA sequences.

### Functional analysis of putative mRNA

The contigs obtained from the assemblies of cDNA reads were aligned to the NCBI nr protein database using BLASTX searches in order to obtain functional annotations. Only hits with an e-value less than or equal to 10^−5^ were considered in the assignments. In order to study mRNA distribution in standardized categories, GO (Gene Ontology) terms for each transcript were retrieved with the BLAST2GO software [31]. The number of reads associated to each contig was considered as an indicator of the corresponding transcript expression level.

In order to detect significantly overrepresented or underrepresented GO categories in a mRNA subset, a Fisher two-tailed enrichment test was performed, using an e-value of 0.05 as threshold.

## Results

### Taxonomic diversity of resin-tolerant microbial communities

The main goal of this work was to study the diversity of microorganisms able to degrade terpenes, the main components of pine tree resin. Galls and environmental resin suspensions were cultured in a selective medium with resin as the main carbon source, and the microbial communities able to grow in such medium were then identified by high-throughput sequencing. After 14 days of incubation, a lawn of microbial colonies was observed in those Petri plates with low (0.05% w/v) to slightly high (0.2% w/v) concentration of resin, whereas a significantly lower number of colonies was observed in media with high resin concentration (0.4% and 0.8% w/v). Some of the plates were clearly dominated by a fluorescent bacterial species. This was particularly striking in plates where galls suspensions had been inoculated and, interestingly, no fungal colonies were observed in these plates (data not shown). The cultivable, resin-tolerant microbial communities associated with environmental resin and galls were pyrosequenced, yielding 721,575 and 660,246 reads, respectively, representing approximately 280 Mb of DNA sequence for each community. Prior to taxonomic and functional analysis of the data, all reads were *de novo* assembled, and ORFs predicted and extracted from the assemblies. The results of these procedures are summarized in Table 1. The assembly of gall sequences exhibited a lower number of contigs (2,613) compared to the 14,102 obtained for the environmental resin, and also longer contigs (Table 1).

**Table 1:** Summary of the sequencing, assembly, and ORF prediction statistics for the microbial communities cultivated from environmental resin and galls.

	Resin	Galls
Megabases generated	283.79	279.56
Number of reads	721,575	660,246
Average read length	393.3	423.42
Number of contigs	14,102	2,613
Max. contig length (bp)	87,262	793,584
Average contig length (bp)	1,504±2,682	2,856±20,534
Mean coverage	12.91	34.72
N50	4,956	152,572
N90	773	1,040
Number of unique proteins	19,136	5,868
Number of unique non-coding RNAs	1, 345	246

**Figure 1:**
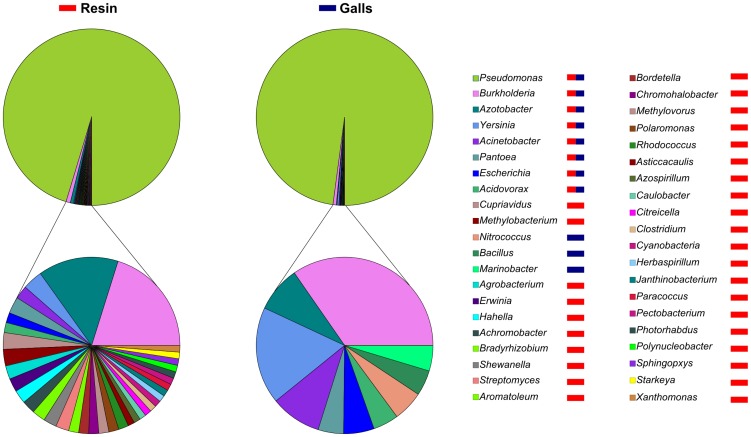
Relative abundance of bacterial genera in the communities cultivated from environmental resin and galls. Horizontal bars indicate the presence of a particular genus in environmental resin (red) or galls (blue).

The taxonomic binning of the samples performed by the MG-RAST server (using the pyrosequencing reads) and the taxonomic composition inferred by an alternative *ad hoc* method (using the ORFs predicted from the assemblies) yielded similar results. *Pseudomonas* proved the most abundant genus in both samples (Figure 1). Taxonomic assignments on the basis of BLASTX searches suggested that *Pseudomonas fluorescens* was the predominant species in both cases. In parallel, sequences from the house-keeping genes *16 S*, *rpoD*, and *gyrB* were retrieved from the sequencing data, and a phylogenetic reconstruction was carried out in order to confirm BLASTX-based taxonomic assignations. Only a single copy of these genes belonging to the genus *Pseudomonas* was found in the galls metagenome, whereas two copies were detected in the environmental resin metagenome. One of the copies of each gene, the most represented in terms of number of reads, was more than a 99% identical to the single copy found in galls, suggesting that they might belong to the same species. The phylogenetic analysis based on these house-keeping genes indicated *Pseudomonas abietaniphila* rather than *Pseudomonas fluorescens* as the closest match for the most abundant –and unique- *Pseudomonas* species in galls samples, and the presence of at least two different *Pseudomonas* species in the microbial communities associated with environmental resin (Figure S2). The incongruence between BLASTX searches and the phylogenetic analysis performed with house-keeping genes is likely a consequence of the lack of complete *P. abietaniphila* genomes in the databases used in the analysis.

Besides *Pseudomonas*, ten different genera able to grow on resin medium were identified from gall samples: *Burkholderia*, accounting for 35% of the remaining sequences; *Yersinia*, with 18%; *Acinetobacter*, 9.3%; *Azotobacter*, 8.4%; and *Pantoea*, *Escherichia*, *Acidovorax*, *Nitrococcus*, *Bacillus*, and *Marinobacter*, which were represented with about 5% of sequences each (Figure 1). The diversity of potential terpene-degrading bacteria found in galls was lower than that associated with environmental (pine tree-associated) resin, where a total of 38 different genera were detected. *Burkholderia* and *Azotobacter* were, besides *Pseudomonas*, the two most abundant taxa (21% and 15% of the ORFs not belonging to *Pseudomonas*). Among the remaining genera, only *Yersinia*, *Acinetobacter*, *Pantoea*, *Escherichia*, and *Acidovorax* were also found within the community isolated from galls (Figure 1), with the other 28 genera being exclusive of environmental resin.

The rarefaction curves obtained for each community suggested that the difference between environmental resin and galls in terms of taxonomic diversity is rather high, since an increase in the number of sequences analyzed did not result in saturation of the number of taxa identified in the case of environmental resin (Figure S3). It has to be noted that the taxonomic diversity of environmental resin and, in particular, the diversity of the microbial communities associated with galls might be underrepresented due to the overwhelming abundance of a single *Pseudomonas* species, which accounts for 85% and 95% of the total ORFs found in the cultivable communities of environmental resin and gall samples, respectively.

Under our experimental conditions, cultivable fungal species were rare, particularly in the gall samples, where only 195 out of the 99,509 BLASTX hits corresponded to fungi. This was in accordance with the inhibition of fungal growth observed in those Petri dishes dominated by a fluorescent species (putatively, *Pseudomonas* sp.). In the case of environmental resin, where a similar number of total BLASTX hits were obtained, fungal sequences were three-fold more abundant (Figure S4). All the putative taxonomic assignations in the culturable pools from both gall and environmental resin samples corresponded to ascomycetes, with the only exception being that of the class Tremellomycetes, detected in environmental resin, belonging to basidiomycetes. As shown in Figure S4, the diversity of fungal classes found in environmental resin was higher compared to resin-rich galls, where only sequences belonging to Dothideomycetes, Eurotiomycetes, Leotiomycetes, and Sordariomycetes were detected.

### Functional diversity and metabolic reconstruction

The functional study of the sequencing data was carried out by assigning each one of the ORFs found to a COG category and a KO (KEGG Orthology) identifier. This yielded a very similar distribution of COG categories in both environmental resin and gall-associated microbial communities (Figure S5), with amino acid metabolism, energy production, and translation being the most represented functional groups in the metagenomes. Most metabolic pathways were also common to both environmental resin and galls (Figure S5). This might be a consequence of the high proportion of sequences belonging to the same species (*Pseudomonas* sp.) in both samples.

The complete sequence of a diterpene-degradation cluster, first reported by Martin and Mohn [38] in *P. abietaniphila* BKME-9, a natural isolate from pulp mill effluents, was found in the gall pool, whereas several partial copies of all the genes were detected in the case of environmental resin (Table S1). The analysis of the 11 kb sequence of the cluster found in galls revealed that it belonged to *Pseudomonas* sp. (hereafter *Pseudomonas abietaniphila* strain PS, taken after *Pinus sylvestris*), the most abundant species in both metagenomes, and displayed the same gene synteny as *P. abietaniphila* BKME-9. On the other hand, several copies of most of the genes involved in pinene degradation (KEGG Pathway 00903) were found in both samples. Sequences encoding particular genes of the acyclic terpene degradation pathway were also detected (Table S1).

### Characterization of the terpene-degrading ability of PS and several resin-associated fungal strains

**Figure 2:**
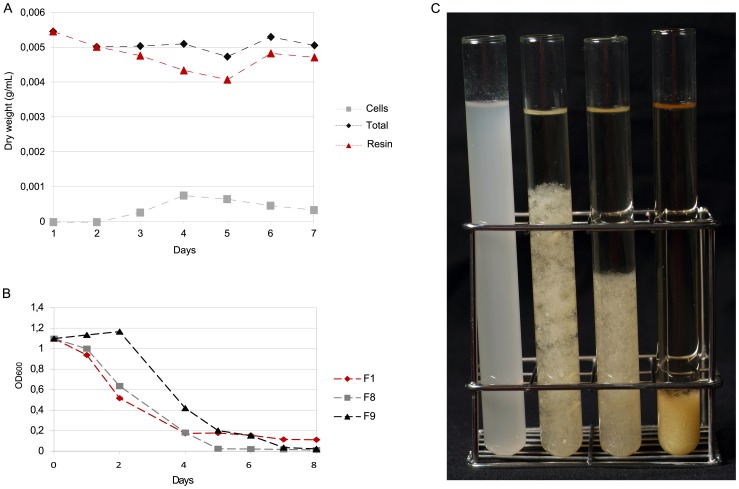
Resin degradation by *Pseudomonas abietaniphila* strain PS and the fungal strains isolated from environmental resin. A) Bacterial and total dry weight of RM cultures of strain PS and evolution of the estimated amount of resin in the medium. B) Variation in resin content in RM cultures of different fungi isolated from resin, estimated from optical density of the medium (OD_600_), as described in Methods S1. C) Resin colloid removal by fungal isolates grown for 7 days compared to a non inoculated RM control (from left to right, control, F9, F8, and F1 cultures).

As expected from the abundance of PS found in the bioinformatic analysis, this strain, which we isolated and grew on resin-containing medium, exhibited good performance in terms of resin degradation. The resin content (estimated as described in Methods S1) during the exponential phase of a PS culture in RM decreased from 5.4 g/L to 4.1 g/L after 4 days, indicating a degradation of the resin originally present in the medium by nearly 25% (Figure 2A). After 5 days, when the culture reached the stationary phase, the amount of resin could not be estimated properly, since the number of viable cells significantly differed from the total number of cells. The growth of PS in RM was accompanied by the secretion of a fluorescent compound, probably one of the siderophores usually produced by different *Pseudomonas* species under conditions of iron starvation [32], [33].

Several fungi able to grow on RM were isolated from environmental resin samples, and pure cultures were inoculated in liquid medium. Three strains, identified as *Aspergillus terreus* (isolate F1), *Aspergillus flavus* (isolate F8), and *Penicillium decumbens* (isolate F9) through 18S rDNA sequencing, were subjected to further characterization in terms of terpene degradation. We managed to follow the changes in resin content in RM cultures of these fungi using a simple method based on measuring optical density of the medium, as described in Methods S1. The number of resin colloids dramatically decreased as fungi grew in the medium, resulting in decreasing optical densities reaching nearly zero after 5 days of cultivation, in the case of isolate F8; and 7 days, in the case of isolates F1 and F9 (Figure 2B). As shown in Figure 2C, RM broth became virtually transparent as a consequence of the degradation of resin colloids and setting of fungal hyphae. The formation of mycelium spheres was observed in all the cultures (Figure S6).

**Figure 3:**
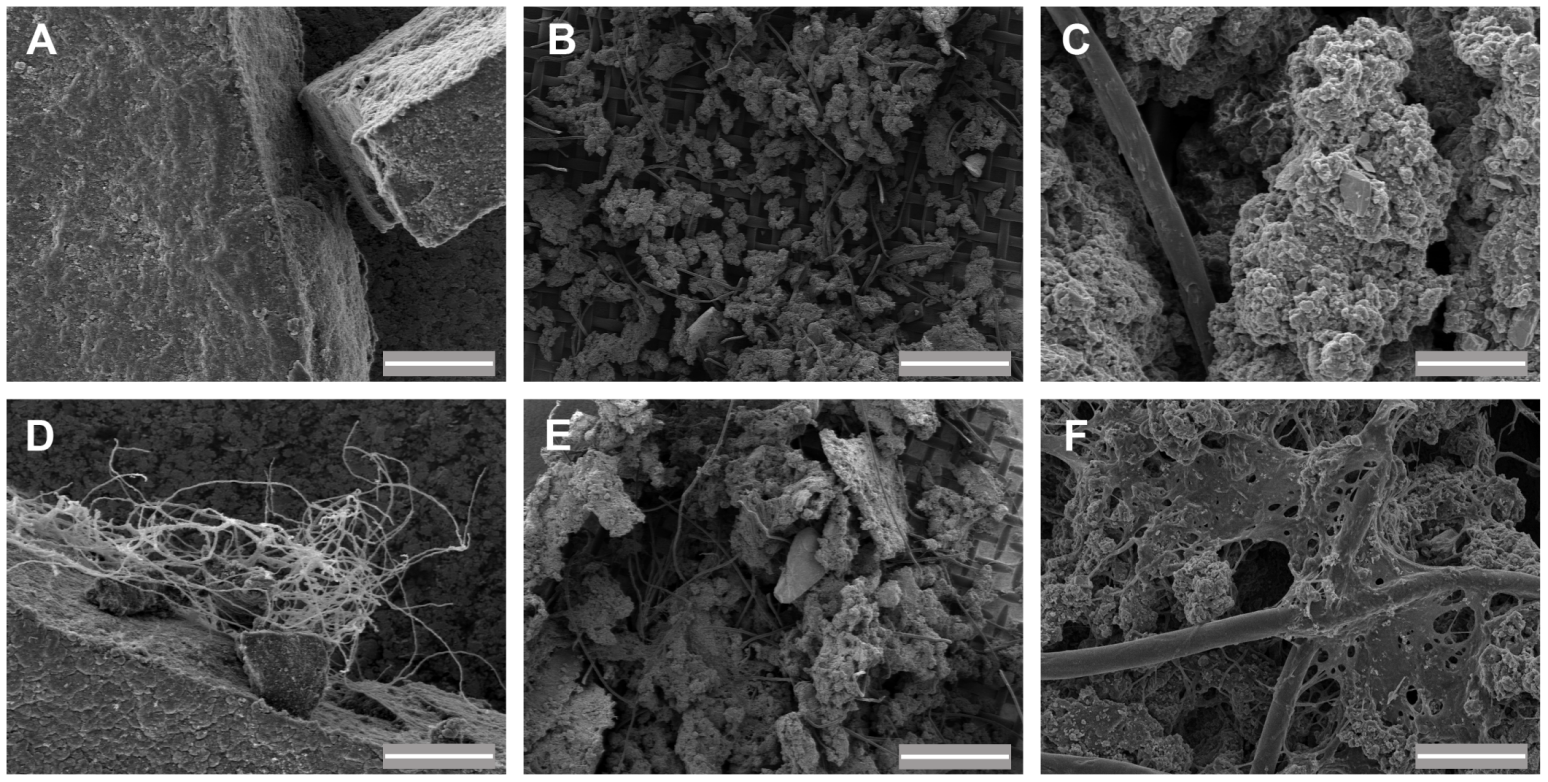
SEM images of latex and rubber degradation performed by fungi. Latex (A and D) and rubber (B, C, E, and F) were used as the sole carbon source in the selective media. A, B and C show non-inoculated control media, whereas 15-day-old cultures of isolates F9 and F1 are shown in subfigures D and E–F, respectively. A, C, D and F scale bars = 50 µm; B, E scale bars = 500 µm.

**Figure 4:**
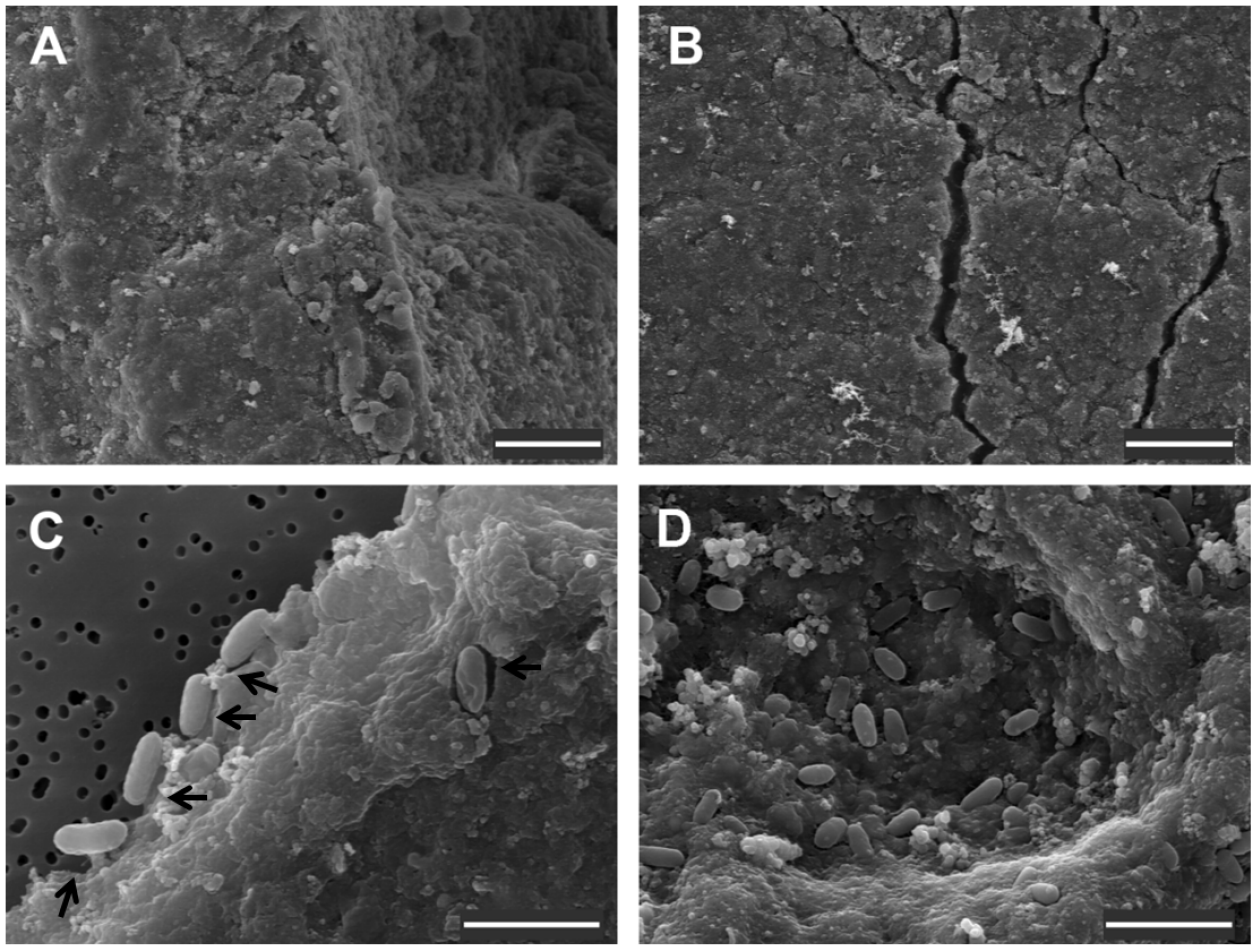
SEM images of latex degradation performed by *Pseudomonas abietaniphila* strain PS. Particles from (A) a non-inoculated latex-containing medium; and a 15-days (B) and a one-month culture (C and D) of strain PS in the same medium are shown. Arrows indicate the cell-shaped niches formed on the latex surface. A and B scale bars = 10 µm, C and D scale bars = 2 µm.

The ability of the four selected microbial strains to degrade other terpene-based materials was tested in minimal media with latex and rubber as the sole carbon sources. All the strains proved able to grow on latex. F1, F8 and F9 fungal hyphae grew in close association with the latex particles, as observed by scanning electron microscopy (SEM) (Figure 3D). *Pseudomonas* strain PS also grew in the latex-containing medium, producing a fluorescent substance [32], [33]. The growth of this strain correlated with the appearance of cracks on the surface of many of the latex particles (Figure 4B). Interestingly, after one month of growth under relatively strong agitation (250 rpm), PS cells exhibited a clear attachment pattern to the latex particles, being virtually embed into the latex structure as a consequence of *in situ* degradation around cells, which resulted in cell-shaped cavities or niches (Figure 4C and 4D). All isolates, with the only exception being PS, also grew in the rubber-containing medium displaying, again, an association with rubber particles. F1 formed dense biofilms covering the rubber and fungal hyphae (Figures 3E and 3F).

### Fungal growth inhibition by strain PS

**Figure 5:**
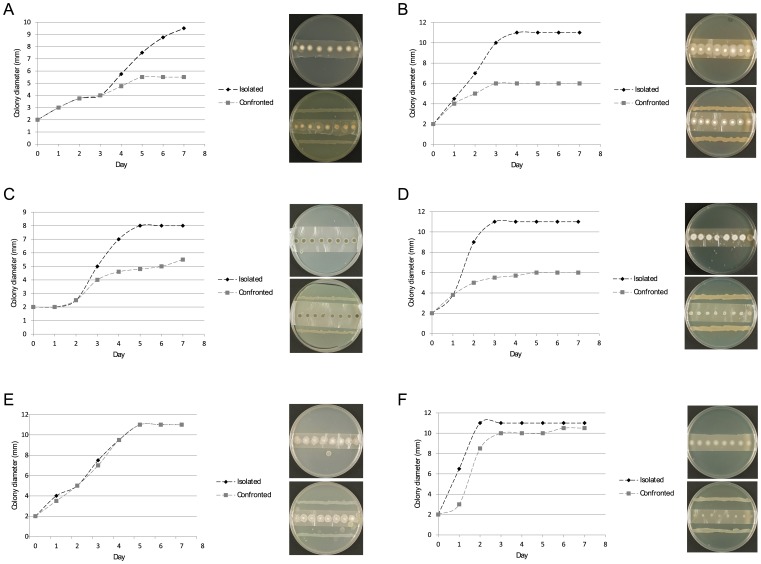
Colony size of three fungal isolates during confrontation assays with strain PS. A and B, F1; C and D, F8; and E and F, F9. The experiments were carried out on RM (A, C, and E) and LB (B, D, and F) medium. Colony size measured in mm. Pictures of particular experiments were taken after 3 and 5 days in the case of LB and RM media, respectively.

PS proved able to inhibit the growth of isolates F1 and F8 in confrontation assays regardless of the medium used (RM or LB). As shown in Figure 5, a reduction of almost 40% was observed in the diameter of F1 colonies on RM, and they stopped growing after 5 days of confrontation. Similar results were obtained on LB, with a reduction of 46% in colony diameter and a halt in fungal growth after 3 days. The inhibition of F8 displayed the same pattern, with a reduction of 37% and 46% in colony diameter on RM and LB, respectively. In the case of F9, no significant differences in terms of colony size were found on RM, whereas a slight inhibition was observed in the confrontations performed on LB. Control experiments carried out with *E. coli* and with a filter-sterilized supernatant of PS cultures, showed no fungal growth inhibition in any case (data not shown).

**Figure 6:**
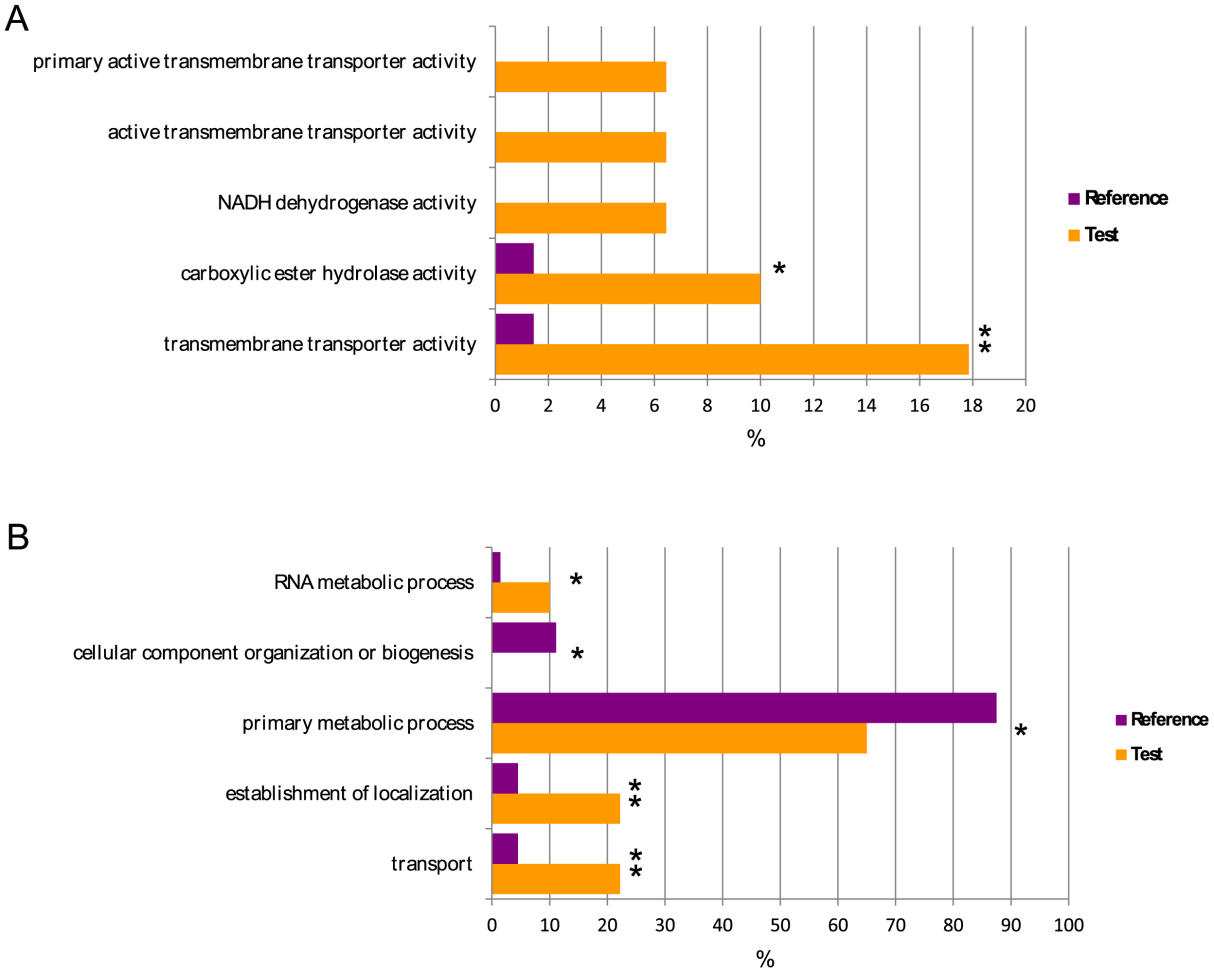
Relative abundance of selected GO terms in the transcriptome of fungal isolate F1. GO terms corresponding to molecular functions (A) and cellular processes (B) are shown for F1 confronted with PS (reference, purple bars), and for the subset of genes not detected in the transcriptome of F1 grown isolated (test, orange bars). Asterisks indicate statistically significant differences for p-value<0.05 (**) and p-value<0.1 (*).

The transcriptomes of F1 grown both isolated and confronted with strain PS on LB (showing the highest fungal inhibitions, as shown in Figure 5) were pyrosequenced in order to check whether transcriptional changes occurred in the fungi as a response to PS. The sequencing statistics of these transcriptomes are shown in Table S2. The analysis of nearly 300 and 200 protein-coding transcripts corresponding to the fungus grown isolated and confronted with PS, respectively, revealed that both transcriptomes had a similar global distribution of GO terms in their mRNAs. However, further analysis revealed that 44 mRNAs of the confronted fungus were not detected in the transcriptome of the isolated fungus (Data S1). A two-tailed Fisher enrichment test performed with this subset of 44 genes against the whole transcriptome of the confronted fungal strain revealed that GO terms involved with transmembrane transportation were significantly overrepresented in the subset (p-value <0.05) (Figure 6).

## Discussion

From the Spanish mountains to the Far East of Russia, and from the Arctic Circle to the Mediterranean, we find *Pinus sylvestris*, one of the most widespread tree species on Earth. The economic and ecological relevance of *P. sylvestris* forests is undeniable, representing major sources of wood and pulpwood, the processing of which generates terpenes as main contaminants of industrial wastewater [15], [16].

The diversity of microorganisms associated with *P. sylvestris* resin and selected on resin-containing medium as reported for the first time here can be likened to a biological arsenal with terpene-degrading ability. Albeit influenced by the composition of the resin-containing medium or the antimicrobial compounds secreted by the isolates (in particular, by *Pseudomonas abietaniphila* strain PS), we report here the highest diversity of terpene-degrading microorganisms described to date. Previously, surveys of microorganisms able to degrade individual terpenes have been carried out in environments such as pulp mill industry effluents [34], sequencing batch bioreactors [35], hydrocarbon-contaminated soils [36], or forest soils [37]. Those works were based on culture-dependent techniques and PCR-based identifications, and reported the ability of several bacterial species belonging to the genera *Pseudomonas*, *Burkholderia*, and *Cupriavidus* to degrade specific resin acids such as dehydroabietic or isopimaric acid (diterpenes). All these genera were detected in our resin-selected samples, and proved to be moderately to highly abundant.

We further characterized particular microbial strains to assess their potential applications for bioremediation of terpene-contaminated environments. Strain PS proved able to both tolerate and degrade significant amounts of resin. Our results indicate that PS degraded nearly 1.5 g of resin per liter after 4 days of exponential growth. This is probably an underestimation of the real value, since the protocol we used to determine the resin content only took into account the amount of resin that is converted into biomass and hence contributes to the increase in dry weight of viable bacteria. The production of any carbonated compound as a result of terpene processing, may also contribute to a decrease in resin content, which suggests that the performance of PS in terms of resin degradation is actually higher than we calculated. On the other hand, different fungal strains isolated from environmental resin samples were also cultivated in resin-containing medium and found to display a dramatic ability to degrade not only resin (Figure 2) but also terpene-based materials such as latex gloves or non-vulcanized rubber. Some of the fungi were able to form a biofilm covered by a dense EPS matrix (Figure 3). These strains may be of interest not only for the bioremediation industry, particularly in the treatment of terpene-contaminated effluents, but they are also potential candidates for the waste management of large-scale disposal materials, such as tire rubber.

The gene content of strain PS was analyzed based on the vast number of sequences obtained, which might cover almost 35% of its complete genome, and a cluster of genes responsible for degradation of diterpenes was detected. This cluster was previously reported in *Pseudomonas abietaniphila* BKME-9 [38] and *Burkholderia xenovorans* LB400 [39], and has recently been detected in other species, whose ability to degrade diterpenes remains unstudied. Case examples of these species are *P. fluorescens* F113 [40] and *P. aeruginosa* 2192 [41]. The cluster found in the selected gall-associated community corresponded to PS, and displayed the same gene synteny as *P. abietaniphila* BKME-9, pointing out, again, that both strains are closely related.

The compatibility of strain PS with other terpene-degrading isolates is also relevant for its application in bioremediation processes. PS proved able to inhibit the growth of different fungi naturally present in resin (Figure 5). Sequencing of the transcriptomes of the fungal isolate F1 (identified as *A. terreus*), grown in isolation as well as under confrontation with PS, revealed changes in the populations of mRNAs involved in transmembrane transportation or coding for proteins with unknown function. Our results are in concordance with a previous transcriptomic analysis of confrontations between *Collimonas fungivorans* and *Aspergillus niger* [30], where moderate transcriptional changes were observed (affecting 0.4% of the transcriptome) in early stages of confrontation, and several genes linked to the fungal cell membrane or coding membrane transporters were up- or down-regulated in response to *C. fungivorans*. As proposed in other works [30], these changes might be linked to nutrient shortage (mainly nitrogen) experienced by the fungi during confrontation, leading to the overexpression of genes involved in nutrient intake.

This is the first holistic and bioprospection-oriented study of microbial communities associated with *P. sylvestris* resin and *R. resinella*-induced galls. The genes, species, and interactions described in this work represent potential tools for several biotechnological processes involving terpenes, and, particularly, for the bioremediation of environments contaminated with these recalcitrant metabolites.

## Supporting Information

Figure S1 **Correlation between contig length and number of reads.** Data corresponding to environmental resin (A) and galls (B) assemblies.
(TIF)

Figure S2 **Phylogenetic analysis of house-keeping genes corresponding to the genus** ***Pseudomonas*** **found in galls and environmental resin.** Sequences from a range of *Pseudomonas* species were retrieved from the NCBI Nucleotide database, and Neighbor Joining trees for *gyrB* (A) and *rpoD* (B) nucleotide sequences were obtained with software MEGA.
(TIF)

Figure S3 **Rarefraction curves obtained for the sequencing data.** The analysis was performed for both environmental resin (red) and galls (blue).after processing the BLASTX results with the software MEGAN, as described in Materials and Methods.
(TIF)

Figure S4 **Abundance and distribution of fungal taxa in environmental resin and gall-associated microbial communities.** The absolute abundance of fungal sequences (expressed as number of BLASTX hits matching fungal sequences) and the relative distribution of fungal taxa are shown.
(TIF)

Figure S5 **Functional reconstruction of the cultivable microbial communities associated with environmental resin and galls.** A) Distribution of annotated genes according to COG functional categories for environmental resin- (red) and gall- (blue) cultivated communities. B) Schematic representation of the KEGG Pathways shared by both samples (blue); and of those found exclusively in environmental resin (green) or galls (red) samples.
(TIF)

Figure S6 **SEM images of typical mycelium spheres.** Spheres were obtained after growing (A) F1 and (B) a resin sample in RM medium for 10 days. A scale bar = 200 µm, B scale bar = 500 µm.
(TIF)

Table S1 **List of genes involved in the degradation of different terpene families detected in resin and gall-associated selected communities.**
(DOCX)

Table S2 **Summary of the sequencing and assembly statistics for F1 transcriptomes.**
(DOCX)

Table S3 **Identification of a selection of fungal strains isolated from environmental resin samples according to 18 S rDNA sequence similarity.**
(DOCX)

Data S1 **List of mRNAs exclusively found in the transcriptome of the confronted fungus.**
(XLS)

Methods S1 **Supporting information on the methods used in this work.**
(DOCX)
