# Supplementary figures and images for: Selecting Microbial Strains from Pine Tree Resin: Biotechnological Applications from a Terpene World
Source: PLoS One. 2014 Jun 27;9(6):e100740. doi: 10.1371/journal.pone.0100740 (PMC4074100; doi:10.1371/journal.pone.0100740)

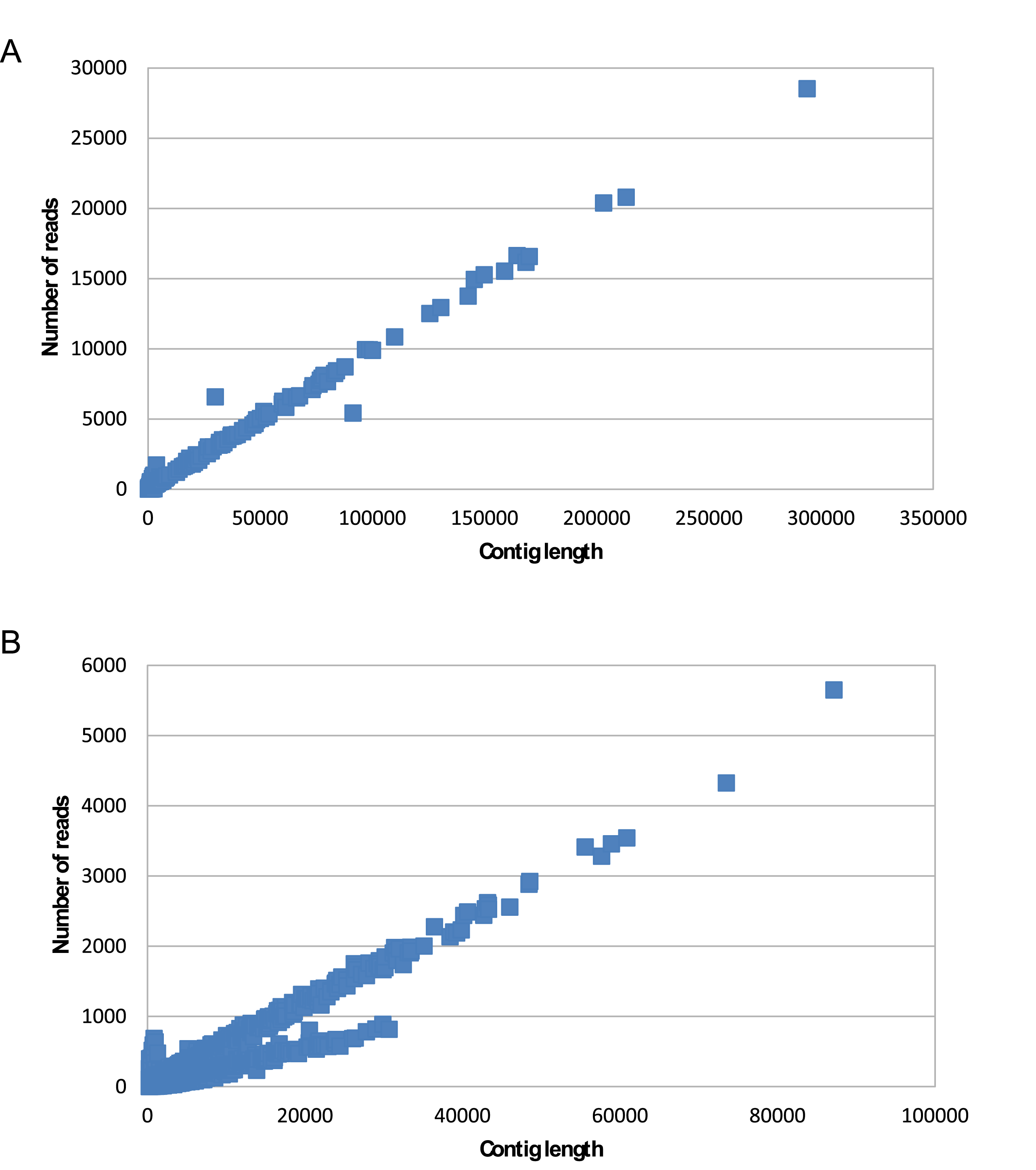

Supplement: Figure S1 — Correlation between contig length and number of reads. Data corresponding to environmental resin (A) and galls (B) assemblies. (TIF) [file pone.0100740.s001.tif]

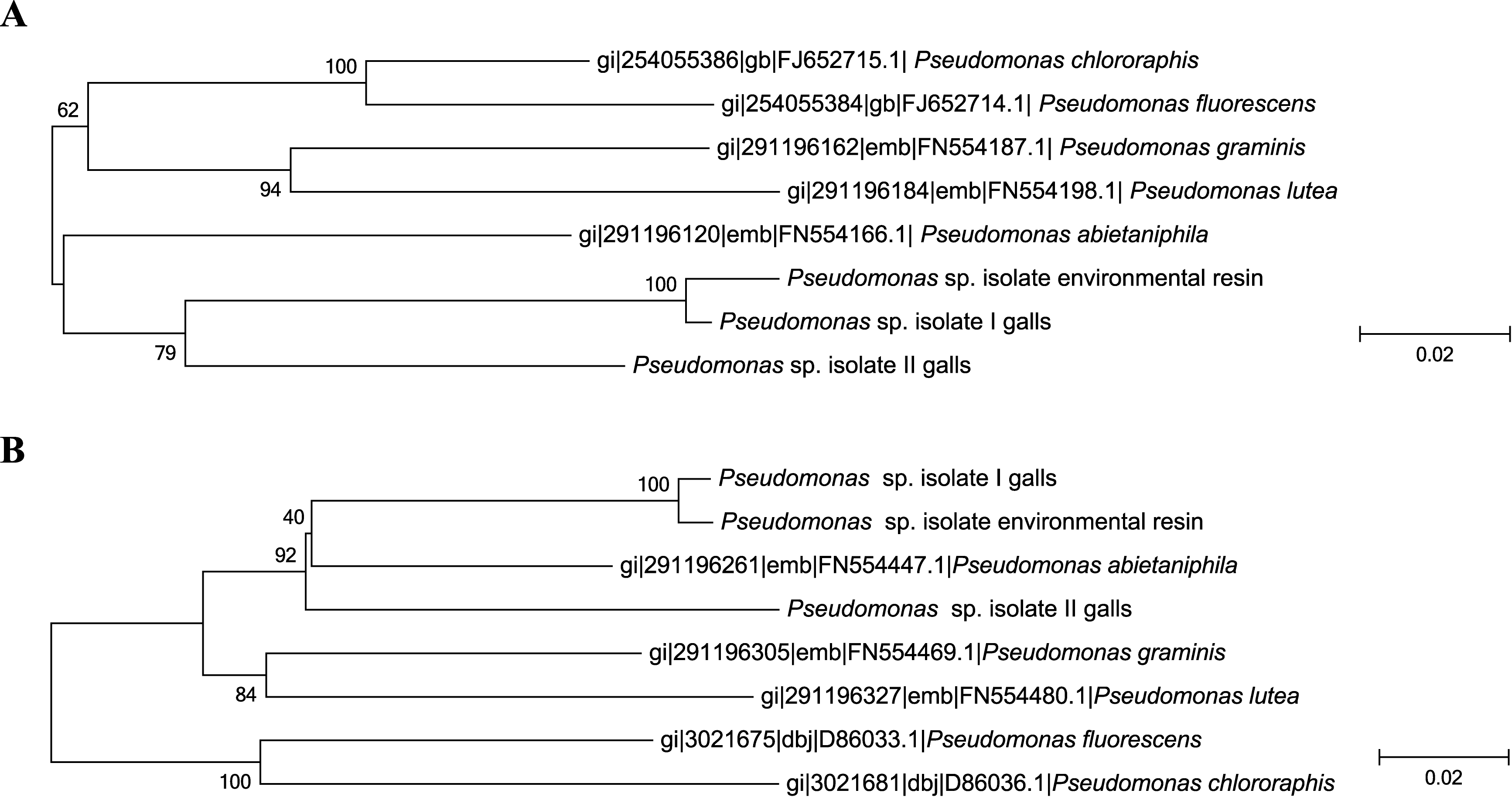

Supplement: Figure S2 — Phylogenetic analysis of house-keeping genes corresponding to the genus Pseudomonas found in galls and environmental resin. Sequences from a range of Pseudomonas species were retrieved from the NCBI Nucleotide database, and Neighbor Joining trees for gyrB (A) and rpoD (B) nucleotide sequences were obtained with software MEGA. (TIF) [file pone.0100740.s002.tif]

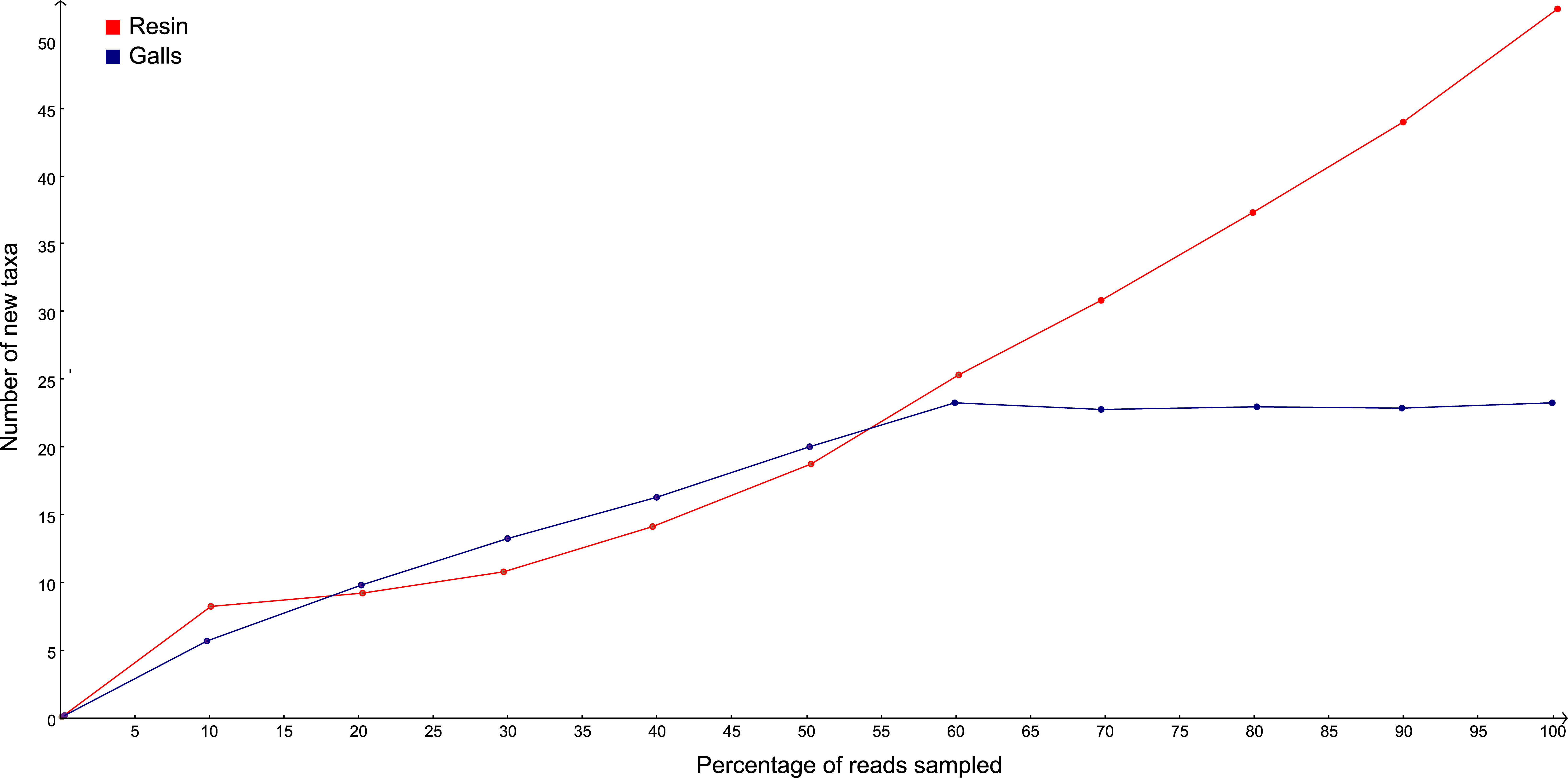

Supplement: Figure S3 — Rarefraction curves obtained for the sequencing data. The analysis was performed for both environmental resin (red) and galls (blue).after processing the BLASTX results with the software MEGAN, as described in Materials and Methods. (TIF) [file pone.0100740.s003.tif]

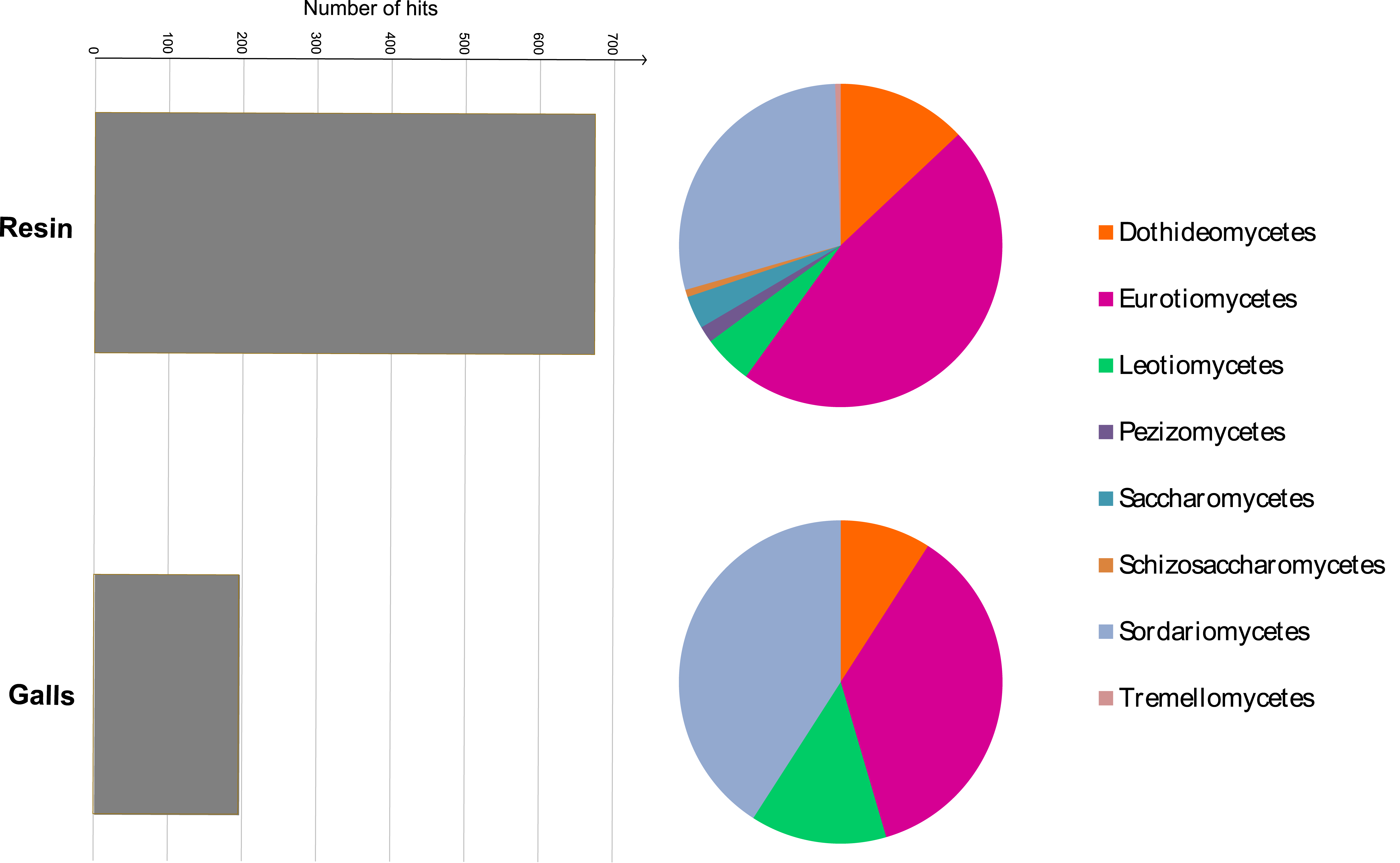

Supplement: Figure S4 — Abundance and distribution of fungal taxa in environmental resin and gall-associated microbial communities. The absolute abundance of fungal sequences (expressed as number of BLASTX hits matching fungal sequences) and the relative distribution of fungal taxa are shown. (TIF) [file pone.0100740.s004.tif]

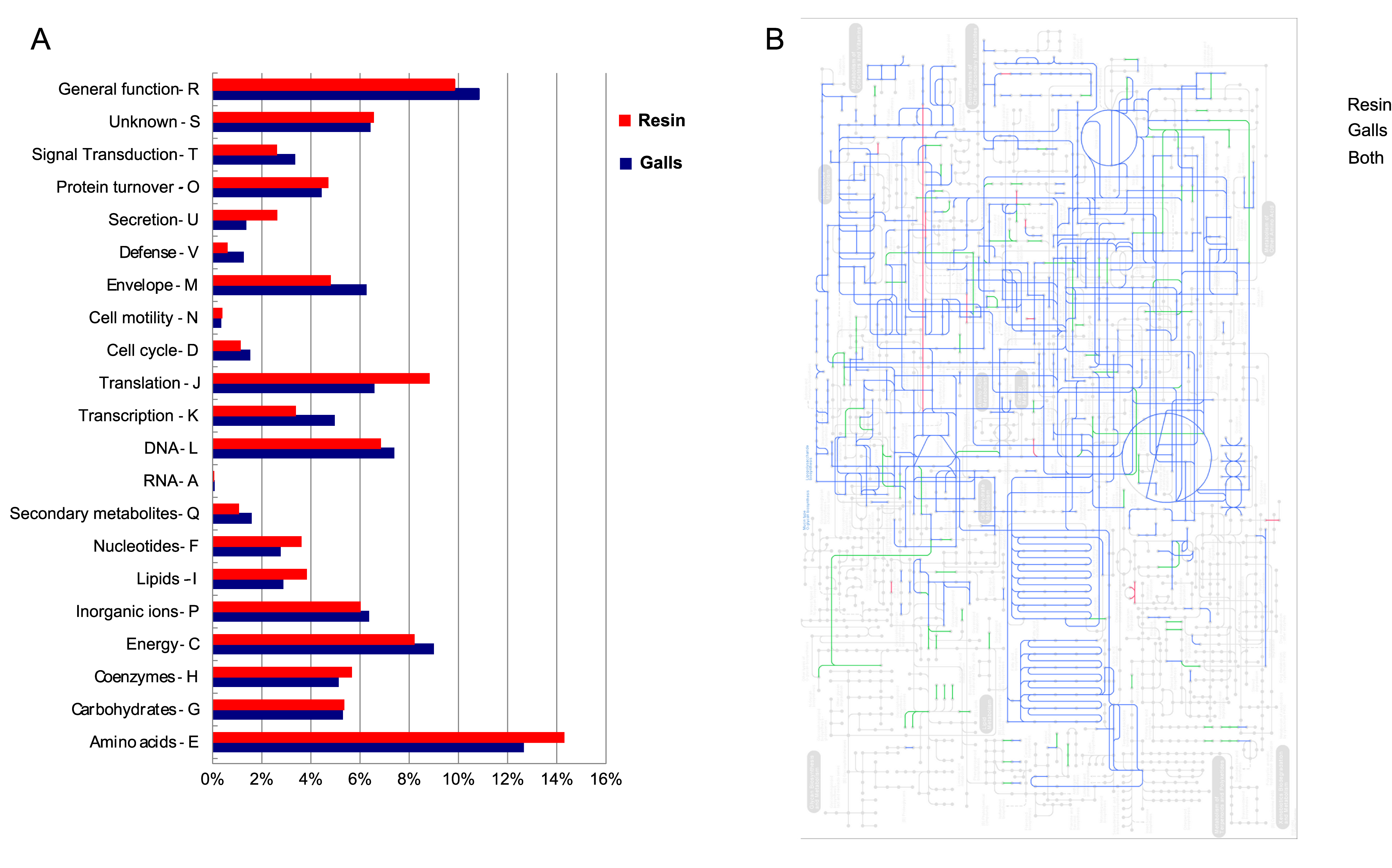

Supplement: Figure S5 — Functional reconstruction of the cultivable microbial communities associated with environmental resin and galls. A) Distribution of annotated genes according to COG functional categories for environmental resin- (red) and gall- (blue) cultivated communities. B) Schematic representation of the KEGG Pathways shared by both samples (blue); and of those found exclusively in environmental resin (green) or galls (red) samples. (TIF) [file pone.0100740.s005.tif]

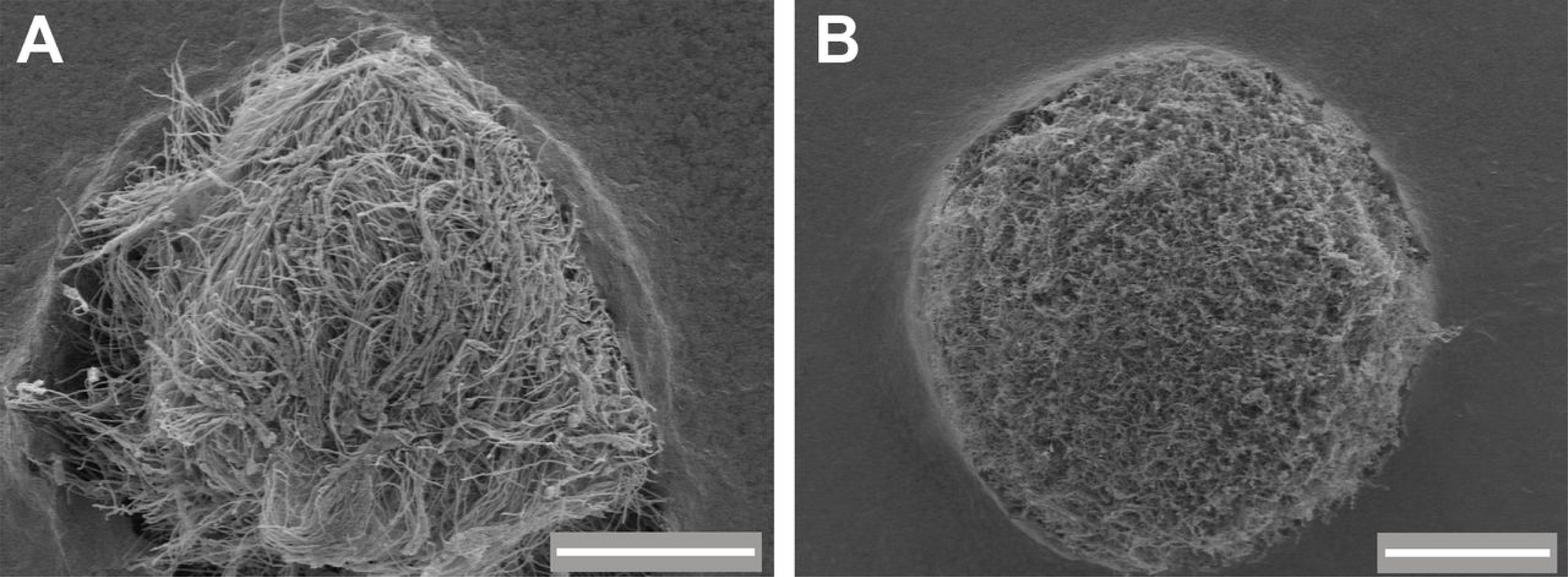

Supplement: Figure S6 — SEM images of typical mycelium spheres. Spheres were obtained after growing (A) F1 and (B) a resin sample in RM medium for 10 days. A scale bar = 200 µm, B scale bar = 500 µm. (TIF) [file pone.0100740.s006.tif]
