# Supplementary material for: Selecting Microbial Strains from Pine Tree Resin: Biotechnological Applications from a Terpene World
Source: PLoS One. 2014 Jun 27;9(6):e100740. doi: 10.1371/journal.pone.0100740 (PMC4074100; doi:10.1371/journal.pone.0100740)
Supplement: Table S1 — List of genes involved in the degradation of different terpene families detected in resin and gall-associated selected communities. (DOCX) [file pone.0100740.s007.docx]

**Supplementary Table 1.** List of genes involved in the degradation of different terpene families detected in resin and gall metagenomes.

|  | **Gene ID** | **Source** | **Description** | **#counts in resin^*^** | **#counts in galls^*^** |
| --- | --- | --- | --- | --- | --- |
| **Pinene (monoterpene) degradation** | K00492 | KEGG Pathway 00903 | α-Pinene monooxygenase | 23 | 1 |
|  | K00155 |  | cis-2-Methyl-5-isopropylhexa-2,5-dienal dehydrogenase | 6 | 1 |
|  | K01913 |  | cis-2-Methyl-5-isopropylhexa-2,5-dienoate-CoA ligase | 2 | 0 |
|  | K01692 |  | cis-2-Methyl-5-isopropylhexa-2,5-dienoyl-CoA hydro-lyase | 33 | 9 |
|  | R06405 |  | 3-Hydroxy-2,6-dimethyl-5-methylene-heptanoyl-CoA dehydrogenase | 1 | 0 |
|  | K00680 |  | 2,6-Dimethyl-5-methylene-3-oxo-heptanoyl-CoA C-acetyltransferase | 29 | 4 |
|  | K01076 |  | 3-Isopropylbut-3-enoyl-CoA thioesterase | 14 | 3 |
|  | K00517 |  | α-Pinene dehydrogenase | 10 | 0 |
|  |  |  |  |  |  |
| **Diterpene degradation** | *ditI* | Martin and Mohn, 2000;  Smith *et al*., 2007 | Dehydrogenase/reductase | 2 | 1 |
|  | *ditA2* |  | β Subunit of the ring-hydroxylating dioxygenase | 3 | 1 |
|  | *ditA1* |  | α Subunit of the ring-hydroxylating dioxygenase | 2 | 1 |
|  | *ditH* |  | Isomerase/decarboxylase | 2 | 1 |
|  | *ditG* |  | Dehydrogenase/reductase | 2 | 1 |
|  | *ditF* |  | Sterol carrier-like protein | 3 | 1 |
|  | *ditR* |  | IclR-type transcription regulator | 3 | 1 |
|  | *ditE* |  | Permease of the major facilitator superfamily | 2 | 1 |
|  | *ditD* |  | Isomerase/decarboxylase | 2 | 1 |
|  | *ditC* |  | Extradiol cleavage dioxygenase | 3 | 1 |
|  | *ditB* |  | Dehydrogenase/reductase | 3 | 1 |
|  | *ditA3* |  | Ferredoxin component of ring-hydroxylating dioxygenase | 3 | 1 |
|  | *ditJ* |  | CoA ligase | 3 | 1 |
|  | *ditK* |  | Transcriptional regulator, TetR family | 3 | 1 |
|  | *ditL* |  | Hypothetical protein | 2 | 1 |
|  | *ditM* |  | Hydrolase | 3 | 1 |
|  | *ditN* |  | 3-hydroxyacyl CoA dehydrogenase | 3 | 1 |
|  | *ditO* |  | Thiolase | 2 | 1 |
|  | *ditP* |  | Conserved hypothetical protein | 0 | 1 |
|  | *ditQ* |  | Cytochrome P450 | 3 | 1 |
|  | *ditR* |  | Transcriptional regulator, IcLR family | 3 | 1 |
|  |  |  |  |  |  |
| **Acyclic terpene utilization** | *atuA* | Forster-Fromme and Jendrossek, 2006 | 3-hydroxy-3isohexenylglutaryl-CoA:acetate lyase | 0 | 2 |
|  | *atuB* |  | Citronellol and citronellal dehydrogenase | 1 | 0 |
|  | *atuC* |  | Geranyl-CoA carboxylase carboxyl transferase subunit | 0 | 1 |
|  | *atuD* |  | Citronellyl-CoA dehydrogenase | 1 | 0 |
|  | *atuE* |  | Isohexenylglutaconyl-CoA hydratase | 0 | 0 |
|  | *atuF* |  | Geranyl-CoA carboxylase biotin-containing subunit | 0 | 1 |

*Only hits with an e-value less or equal than 10^-5^ and an identity percentage great or equal than 60% were considered
