# Supplementary material for: Selecting Microbial Strains from Pine Tree Resin: Biotechnological Applications from a Terpene World
Source: PLoS One. 2014 Jun 27;9(6):e100740. doi: 10.1371/journal.pone.0100740 (PMC4074100; doi:10.1371/journal.pone.0100740)
Supplement: Table S2 — Summary of the sequencing and assembly statistics for F1 transcriptomes. (DOCX) [file pone.0100740.s008.docx]

**Supplementary Table 2.** Summary of the sequencing and assembly statistics for F1 transcriptomes.

|  | **Isolated** | **Confrontated** |
| --- | --- | --- |
| **Megabases generated** | 3.23 | 2.18 |
| **Number of reads** | 20,893 | 15,319 |
| **Average read length (bp)** | 189.93 | 228.27 |
| **Number of isotigs** | 472 | 348 |
| **Average isotig length (bp)** | 382.18 | 339.98 |
| **Average number of reads per contig** | 25.83 | 58.74 |
| **Maximum number of reads per contig** | 699 | 701 |
| **Number of protein-coding isotigs** | 299 | 179 |
