# Supplementary material for: Selecting Microbial Strains from Pine Tree Resin: Biotechnological Applications from a Terpene World
Source: PLoS One. 2014 Jun 27;9(6):e100740. doi: 10.1371/journal.pone.0100740 (PMC4074100; doi:10.1371/journal.pone.0100740)
Supplement: Table S3 — Identification of a selection of fungal strains isolated from environmental resin samples according to 18 S rDNA sequence similarity. (DOCX) [file pone.0100740.s009.docx]

| **Isolate name** | **Best Match** | **NCBI Accesion** | **% identitity** |
| --- | --- | --- | --- |
| F1 | *Aspergillus terreus* strain HDJZ-ZWM-18 | GU227345.1 | 99 |
| F8 | *Aspergillus flavus* strain TPID12 | EU263602.1 | 100 |
| F9 | *Penicillium decumbens* isolate MMH 89-p1 | FR774046.1 | 99 |

**Supplementary Table 3.** Identification of a selection of fungal strains isolated from environmental resin samples according to 18S rDNA sequence similarity.
