# Supplementary material for: Selecting Microbial Strains from Pine Tree Resin: Biotechnological Applications from a Terpene World
Source: PLoS One. 2014 Jun 27;9(6):e100740. doi: 10.1371/journal.pone.0100740 (PMC4074100; doi:10.1371/journal.pone.0100740)
Supplement: Methods S1 — Supporting information on the methods used in this work. (DOCX) [file pone.0100740.s011.docx]

**Supporting Methods**

*18S rDNA amplification and sequencing*

The DNA from fungal isolates was extracted with the Power Soil DNA Isolation kit (MO BIO Laboratories) according to the manufacturer's instructions and directly used as PCR template. A 800 bp fragment of the 18S rDNA genes was amplified by PCR with universal primers EF3 (5′-TCCTCTAAATGACCAAGTTTG-3′) and EF4 (5′-GGAAGGG[G/A]TGTATTTATTAG-3′) (Smit *et al*., 1999). The PCR program was as follows: an initial denaturing step at 95 °C for 300 s, followed by 35 cycles of denaturing, annealing and extension (95 °C, 30 s; 48 °C, 30 s; and 72 °C, 90 s) and a final extension step at 72 °C for 480 s. PCR amplicons were purified by the High Pure PCR Product Purification Kit (Roche Diagnostics GmbH, Mannheim, Germany) and sequencing was carried out with the ABI PRISM BigDye Terminator v3.1 system (Applied Biosystems) on an ABI 3730 automated sequencer. PCR products were sequenced in both senses with the EF3 and EF4 primers. Sequences were verified and both strands assembled using the STADEN package. Sequence taxonomy was attributed with BLASTN searches against the RefSeq database of the NCBI.

*Quantification of resin degradation by fungal isolates and PS*

Fungal isolates able to grow on RM were identified through 18S rDNA sequencing as described above. Each isolate was grown at 30 ºC in sterile Erlenmeyer 100 mL flasks with 50 mL of RM broth containing 0.1% w/v resin and shaken at 250 rpm. Water-insoluble resin formed a white colloidal suspension in the RM liquid medium. As fungi degraded resin particles, the medium became more transparent, in such a way that resin degradation was assessed by measuring the optical density (OD) of the medium at 600 nm. As fungi degraded resin particles, the medium became more transparent, in such a way that resin degradation was assessed by measuring the optical density (OD) of the medium at 600 nm. Fungal hyphae did not influence the OD of the culture, since they were strongly aggregated forming mycelium spheres, which quickly settled as a consequence of biofilm production and orbital shaking (Pirt, 1966).

The resin degradation ability of *Pseudomonas sp*. could not be determined with the method described above because bacterial cells in suspension interfered with the OD measurements, and co-sedimented with resin particles when a centrifugation step was performed. Therefore, resin degradation was estimated with an indirect method. The resin content of the medium was estimated by calculating the difference between the total dry weight of the culture and the dry weight of the cellular fraction. This latter value was calculated from the number of viable bacteria (directly measured by plate counting) after establishing a correlation between the number of colony forming units and the corresponding bacterial pellet dry weight in LB broth. This correlation was assumed to be the same for LB and RM cultures of the same strain.

*Preparation of other terpene-based culture media*

The ability of the isolates described in this work to degrade terpene-based polymers was tested in culture media containing latex and rubber as sole carbon sources. To prepare the latex-containing medium, standard laboratory gloves made of natural latex were ground along with dry ice until small latex laminas of 1-3 mm were obtained. A minimal saline solution, like the one used in the resin-containing medium (RM), was supplemented with 40 g/L of latex laminas and used as broth. In the case of the rubber-containing medium, non-vulcanized rubber was kept at -80 ºC for 30 min and then grated to obtain a fine powder. This rubber powder (20g) was added to 1 L of minimal saline solution. Isolates were grown at 30 ºC in sterile Erlenmeyer 100 mL flasks with 50 mL of these media and shaken at 250 rpm until required.

*Scanning electron microscopy of microbial growth on terpene-based materials*

Little pieces of latex, rubber, or simply fragments of mycelium obtained from cultures of the different isolates were fixed by immersion into paraformaldehyde 2% - glutaraldehyde 2.5 % for more than two hours, then lightly washed with water and preserved in ethanol 70%. These pieces were placed inside microporous specimen capsules (30 μm pore size, available from Ted Pella Inc. product number 4619) immersed in absolute ethanol, following critical point drying in an Autosamdri 814 (Tousimis). The fragments so obtained were then arranged on SEM stubs by silver conducting paint TAAB S269. Pieces were always manipulated under a stereomicroscope Leica MZ9.5 with Dumont forceps number 5. Stubs were examined under a scanning electron microscope Hitachi S-4100. Images were edited with Photoshop CS3 (Adobe).
